# Node-negative colon cancer: histological, molecular, and stromal features predicting disease recurrence

**DOI:** 10.1186/s10020-023-00677-8

**Published:** 2023-06-21

**Authors:** Maud T. A. Strous, Ragna L. A. van der Linden, Audrey L. H. M. Gubbels, Timothy K. E. Faes, Koop Bosscha, Carolien M. Bronkhorst, Maryska L. G. Janssen-Heijnen, Adriaan P. de Bruïne, F. Jeroen Vogelaar

**Affiliations:** 1 Department of Surgery, VieCuri Medical Centre, Tegelseweg 210, 5912 BL Venlo, The Netherlands; 2 Department of Epidemiology, GROW School for Oncology and Developmental Biology, Faculty of Health, Medicine and Life Sciences, Maastricht University, Universiteitssingel 40, 6229 ER Maastricht, The Netherlands; 3 Department of Surgery, Jeroen Bosch Hospital, Henri Dunantstraat 1, 5223 GZ ’s Hertogenbosch, The Netherlands; 4 Department of Pathology, VieCuri Medical Centre, Tegelseweg 210, 5912 BL Venlo, The Netherlands; 5 Department of Pathology, Jeroen Bosch Hospital, Henri Dunantstraat 1, 5223 GZ ’s Hertogenbosch, The Netherlands; 6 Department of Clinical Epidemiology, VieCuri Medical Centre, Tegelseweg 210, 5912 BL Venlo, the Netherlands

**Keywords:** Colon cancer, Node negative, Tumour-stroma ratio, TSR, MSI, BRAF, KRAS, Cancer recurrence, Disease free survival, Survival

## Abstract

**Background:**

Within the group of node-negative colon cancer patients, presumed to have a good prognosis, a significant percentage of patients develops cancer-recurrence. Current high-risk features prove inadequate to select these particular high-risk patients. In the process of tailor-made care and shared decision-making the need to identify these patients grows. In this study we investigate the value of adding molecular markers and the tumour-stroma ratio (TSR) to conventional histological tumour staging methods to improve the selection of high risk patients.

**Methods:**

We retrospectively analysed 201 patients diagnosed with TNM-stage I-II colon cancer and treated by complete oncological resection between November 1st 2002 and December 31st 2012 at the Jeroen Bosch Hospital. Conventional histological tumour staging, BRAF mutations, KRAS mutations, MSI status and TSR were determined. Differences between groups based on TSR and mutation status, in disease free survival were analysed using Cox-Regression analyses.

**Results:**

Poorly differentiated histology (p = 0.002), high-TSR (p = 0.033), BRAF-mutation (p = 0.008) and MSI (p = 0.011) were identified as significant risk factors for cancer recurrence. The risk of recurrence increased in the presence of both a BRAF-mutation and high-TSR compared to the absence of both factors or presence of only one factor (HR = 3.66 BRAF-mt/TSR-low (p = 0.006), HR 2.82 BRAF-wt/TSR-high (p = 0.015), HR = 4.39 BRAF-mt/TSR-high (p = 0.023)). This was also seen in tumours with MSI and high-TSR (HR = 2.46 MSS/TSR-high (p = 0.041), HR = 3.31 MSI/TSR-high (p = 0.045).

**Conclusion:**

Judging by the higher HR for the combination of the prognostic factors TSR and BRAF compared to the HRs of these prognostic factors individually, the prognostication for disease free survival can be improved by determining both TSR and BRAF instead of BRAF alone, as is done in current daily practise. In this study MSI also shows additional value to TSR in the prognostication of disease free survival. Adopting TSR into daily diagnostics will be of additional value next to currently used molecular markers in risk stratification of patients with node negative colon cancer and is therefore advised.

## Background

Oncological surgical resection is the main curative treatment in colon cancer (CC), when indicated in combination with adjuvant systemic therapy. The indication for adjuvant therapy is mainly based on the incidence of lymph node metastasis, as this is one of the most important prognostic factors for oncological outcome (Dutch Guideline 'colonic cancer' 2019). However, the metastatic potential of a tumour is not always predictable by lymph node involvement alone. In spite of curative resection, about 10–25% of node-negative patients develop disease recurrence within 5-years after surgery (Hyslop and Waldman 2013; Kang et al. 2022; Tsikitis et al. 2014). To be able to identify these high-risk node negative patients, who might benefit from adjuvant treatment, various attempts at detecting additional prognostic factors have been made. The American Society of Clinical Oncology (ASCO) has made several recommendations on factors that should be regarded as a high-risk feature. In the most recent recommendations only T4-stage is regarded as a high-risk feature. In case of T4N0-stage the prognosis of a TNM-stage II tumour should be regarded as comparable to TNM-stage III colon cancer. Therefore it is recommended to treat these patients with adjuvant chemotherapy (Baxter et al. 2022). Other factors that could be considered as high-risk feature are the presence of bowel obstruction or perforation, poor histological differentiation, perineural or lymph-angio invasion, grade BD3 tumour budding and inadequate lymph node sampling (< 12 lymph nodes) (Baxter et al. 2022). When present in a TNM-stage II tumour, adjuvant chemotherapy may be offered to the patient. But in spite of this remark, in daily practice adjuvant chemotherapy is only given in 16% of these patients (Brouwer et al. 2018). Even after inclusion of these defined high-risk features, not all patients at high-risk of cancer recurrence are accurately selected.

The biological behaviour of a tumour is not only influenced by cancer cells themselves, but also by the tumour microenvironment or so called intratumoural stroma. Intratumoural stroma has been shown to mediate tumour growth, invasion and metastasis (Conti and Thomas 2011; Wever et al. 2008). It regulates a number of tumour-promoting functions including epithelial-to-mesenchymal transition (EMT), a process wherein cancer cells switch to a mesenchymal phenotype which enhances tumour invasion and metastasis (Lamouille et al. 2014). A marker assessing the amount of intratumoural stroma within a tumour is the tumour-stroma ratio (TSR). This easy, low-cost, and highly reproducible marker has been shown to be of prognostic value for oncological outcome in CC, independent of other known risk-factors (Huijbers et al. 2013; Mesker et al. 2007; Park et al. 2014; Strous et al. 2022).

The content of intratumoural stroma is variable and might be influenced by distinct molecular features of tumour cells (Fridman et al. 2020). For example, the degree of EMT might be influenced by KRAS mutations. This mutation has been shown to activate downstream effectors of the PI3K pathway which, in synergy with TGF-β signalling, induces EMT (Janda et al. 2002; Maffeis et al. 2019). Other mutations in the RAS-RAF-MEK-cascade, as BRAF mutations, also induce EMT by allowing downstream effectors to promote the expression of transcription factors regulating EMT (Maffeis et al. 2019; Lemieux et al. 2009). In contrast, colon tumours with microsatellite instability (MSI) state seem to exhibit impaired EMT (Oh et al. 2016). BRAF mutations have mainly been studied in metastatic disease, in which a BRAF mutation was associated with worse prognosis. Only one study, including non-metastatic node-positive and node-negative patients, showed an association between BRAF mutations and survival in non-metastatic patients, in which the presence of a BRAF mutation predicted worse cancer-free survival (CFS) (Farina-Sarasqueta et al. 2010). Instability of microsatellites, in contrast, has been associated with a better prognosis (Dotan and Cohen 2011; Gelsomino et al. 2016). However, this association has been inconsistent between different studies, as has the prognostic value of KRAS mutations (Gao et al. 2005; Palomba et al. 2016; Roth et al. 2010). Detection of these molecular markers is, in current clinical practise, only indicated when specific cancer treatments are considered and the tumour’s potential resistance against this treatment should be determined. Another reason for detection is to rule out hereditary cancer syndromes (Ezaz and Tapper 2019). As these molecular features are potentially related to both tumour cells and intratumoural stroma content, identification of these molecular features in combination with TSR might improve risk-stratification of node negative patients. With a more adequate risk-stratification we might be able to prevent cancer recurrence by implementing a personalized treatment and follow-up.

In previous research we determined the prognostic value of TSR in this cohort of 201 patients who underwent curative oncological resection for node negative CC (13). With this current study we aimed to assess any adding prognostic value of molecular markers (BRAF, KRAS and MSI) to the prognostic value of TSR in node negative CC. In addition, as molecular mutations in tumour cells seem to affect the intratumoural stroma, we aimed to assess a potential correlation between these molecular markers and the intratumoural stroma derived marker TSR.

## Methods

### Patients and data

The study population consisted of all consecutive patients diagnosed with a TNM-stage I–II primary CC, and treated by complete oncological resection between November 1st 2002 and December 31st 2012 at Jeroen Bosch Hospital. The following patients were excluded: patients developing metastatic disease within 3 months after surgery (as those metastases were considered present at time of surgery), patients with a non-adenocarcinoma, patients receiving adjuvant chemotherapy, and patients of whom tumour tissue was missing or insufficient for re-assessment. Demographic and clinical data of included patients were obtained from their medical records and combined with data from the Netherlands Cancer Registry (NCR) that collects data on all newly diagnosed cancer patients in the Netherlands. The tumour-node-metastasis (TNM) classification was used for staging of the primary tumour, according to the edition valid at time of cancer diagnosis. Comorbidities were registered according to a slightly modified version of the Charlson Comorbidity index (Charlson et al. 1987).

### Histopathological analyses of the tumour-stroma ratio

**Fig. 1:**
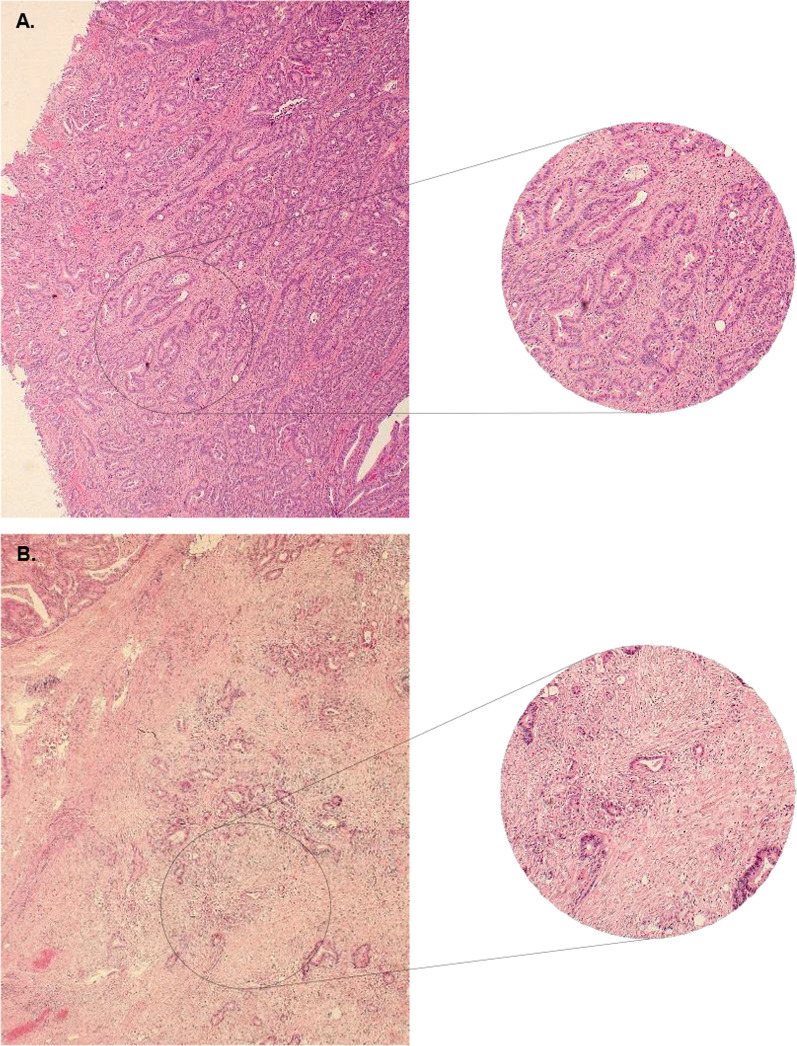
Histological image of a haematoxylin and eosin (H&E)-stained tissue section of a TSR-low (**A**) and TSR-high (**B**) tumour

The amount of intratumoural stroma was estimated by microscopic analysis of 4 µm haematoxylin and eosin (H&E)-stained tissue sections of the primary tumour using a scoring technique described by van Pelt et al. (2018). Using an ×2.5 or ×5 objective, the area with the largest amount of intratumoural stroma was selected. Then, an ×10 objective was used to select an area with both intratumoural stroma tissue and tumour cells, where tumour cells were present at all quadrants of the selected image field’s border. Here the tumour-stroma ratio (TSR) was estimated in a range from 10 to 90 percent per 10 percent increment. A cut-off value of 50 percent stroma was set to categorize patients as TSR-low (≤ 50%) or TSR-high (> 50%), as determined in earlier research to be most discriminative (Park et al. 2014). Figure 1 shows a histological image of a TSR-low and a TSR-high tumour.

### Molecular analyses

The method for molecular analyses in included patients was described in detail in the original articles (Linden et al. 2020; Vogelaar et al. 2016). The results of microsatellite analysis, KRAS mutation analysis and BRAF mutation analysis were selected. Microsatellite instability was detected using the mononucleotide repeat BAT26 marker by means of real-time PCR. With the use of ABI3100 (applied Biosystems) and the GeneMapper 4.0 software package products sizes were analysed. KRAS mutations were detected in exon 2, 3 and 4 using PCR high resolution melting (HRM) followed by direct sequencing. For the BRAF gene the V600 mutation was detected with the use of real-time PCR. Sequences were evaluated with the Sequencing Analysis 5.3.1. Software.

### Endpoints and definitions

The primary endpoint of this study was disease free survival (DFS). DFS was defined as the time in months between date of surgery and date of cancer recurrence (defined as first date of either radiologic or pathologic diagnosis of local tumour recurrence or metastasis of colon cancer), or date of last follow-up (with a maximum of 5-years). Standard follow-up examinations were performed using the Dutch oncological follow-up guidelines for colon cancer after curative resection up to 5 years after surgery (Ezaz and Tapper 2019). Patients dying without cancer recurrence were censored on their day of death.

### Statistical analyses

Data was analysed using IBM SPSS Statistics, version 25.0 (IBM Corp, NY, Armonk, USA). Descriptive statistics were performed to provide an overview of the study population divided into groups based on TSR. Continuous variables were expressed as means ± SD or median with interquartile range when not normally distributed; categorical variables were shown as counts and percentages. Continuous variables were compared between groups using unpaired t-tests, and categorical variables were compared using Chi-square statistics or Fisher’s exact test, as appropriate. The prognostic association between TSR and DFS was analysed by univariable and multivariable cox-regression analysis, while adjusting for other prognostic variables. Then, TSR was combined with BRAF- and KRAS-mutation state and microsatellite (in)stability, after which the prognostic association of these combinations was analysed by univariable and multivariable cox-regression analysis, also adjusting for other prognostic variables. Survival curves for disease free survival were plotted using Kaplan–Meier curves. Variables included for adjustment were selected if they showed a p-value of < 0.05 in univariate analysis. Those included patient demographics (age, gender, comorbidities identified at admission according to modified Charlson Comorbidity Index), tumour characteristics (tumour stage, localisation, differentiation and lymph-angio invasion) and treatment characteristics (type and setting of surgery). A two-tailed p-value < 0.05 was considered significant in all analyses.

## Results

After exclusion of 12 patients of whom tumour tissue was missing or insufficient for assessment, and 2 patients presenting with metastasis within 3 months after surgery, 201 patients who underwent curative oncological resection for a TNM I–II colon carcinoma remained. Median age of the study population was 73 years (IQR 65–79), and 117 patients (55.5%) were male. Most patients had pathological stage II disease (n = 169, 84.1%). Cancer recurred in 33 patients (16.4%) during a median follow-up of 60 months (IQR 32–60).

Tumours of 58 patients (28.9%) were classified as TSR-high (> 50%). These tumours were more likely to comprehend the high-risk feature lymph-angio invasion compared to TSR-low tumours, but not a poor differentiation-grade (Table 1). The number of T4 stage tumours in this series was too small to comment on differences of this feature between TSR-groups.

**Table 1:** Baseline characteristics of the study population

	TSR-low	TSR-high	p-value
Age*	72 (65–80)	73 (65–77)	0.743
Gender, n (%)			0.864
Male	77 (53.8)	32 (55.2)	
Female	66 (46.2)	26 (44.8)	
Comorbidities^#^, n (%)			0.402
0	35 (35.7)	15 (38.4)	
1	31 (31.6)	10 (25.6)	
≥ 2	32 (32.7)	14 (35.9)	
Surgery, n (%)			0.103
Elective	137 (95.8)	51 (87.9)	
Acute	6 (4.2)	7 (12.1)	
Tumour localisation, n (%)			0.845
Right colon	74 (51.7)	31 (53.4)	
Left colon	66 (46.2)	26 (44.8)	
pT-stage, n (%)			─
T1	4 (2.8)	0 (0.0)	
T2	18 (12.6)	8 (13.8)	
T3	121 (84.6)	49 (84.5)	
T4	0 (0.0)	1 (1.7)	
TNM stage, n (%)			0.774
I	22 (15.4)	8 (13.8)	
II	121 (84.6)	50 (86.2)	
Differentiation, n (%)			0.816
Well/Moderate	124 (86.7)	51(87.9)	
Poor/Undifferentiated	19 (13.3)	7 (12.1)	
Lymph-angio invasion, n (%)			0.023
No	123 (86.0)	42 (72.4)	
Yes	20 (14.0)	16 (27.6)	
BRAF, n (%)			0.721
Wildtype (wt)	117 (82.4)	49 (84.5)	
Mutant (mt)	25 (17.6)	9 (15.5)	
KRAS, n (%)			0.251
Wildtype (wt)	88 (61.5)	40 (70.2)	
Mutant (mt)	55 (38.5)	17 (29.8)	
Microsatellite, n (%)			0.685
Stable (MSS)	113 (80.1)	45 (77.6)	
Instable (MSI)	28 (19.9)	13 (22.4)	

*Non-normal distributed data presented as median with interquartile range

^#^Abdominal aortic aneurysm, COPD, cerebrovascular accident, peripheral vascular disease, congestive heart failure, mitral valve leakage, cardiovascular disease, peptic ulcer, diabetes mellitus, solid carcinoma (breast, prostate, uterus, cervix, bladder), multiple myeloma and chronic hepatitis

### TSR and molecular markers

BRAF- and KRAS mutations were found in 34 (17.9%) and 72 patients (35.8%), respectively. Tumours of 41 patients (20.4%) were MSI. Tumours with BRAF-mutations were more often right-sided (91.2% versus 45.0%, p < 0.001), and more often poorly differentiated or undifferentiated (35.3% versus 8.4%, p < 0.001), as were MSI tumours (87.8% versus 43.5%, p < 0.001 and 39.0% versus 6.3%, p < 0.001). In MSI tumours a BRAF mutation was present in 68.3% (n = 28, p < 0.001). Presence of a KRAS mutation in a MSI tumour was less likely, 15% in MSI versus 41% in microsatellite stable (MSS) tumours (p = 0.009). No statistically significant association between the intratumoural stroma derived marker TSR and the molecular markers BRAF (p = 0.765), KRAS (p = 0.150) and MSI (p = 0.611) was found (Table 1).

### Survival

Cancer recurred more often in patients with a TSR-high tumour compared to those with a TSR-low tumour, although not significant (14 (24.1%) versus 19 (13.3%), p = 0.060). Both univariable and multivariable analyses showed that DFS was significantly lower in patients with TSR-high tumours compared to TSR-low tumours (univariable HR = 2.12 (CI 1.06–4.23, p = 0.033); multivariable(adjusted for BRAF status and tumour differentiation grade)HR = 2.19 (CI 1.09–4.42, p = 0.028); multivariable (adjusted for microsatellite status and tumour differentiation grade) HR = 2.20 (CI 1.10–4.44, p = 0.027)) (Table 2).

**Table 2:** Univariable and multivariable analyses of associations of TSR and other factors with disease free survival

	Univariable		Multivariable		Multivariable	
	HR (95% CI)	p-value	HR (95% CI)	p-value	HR (95% CI)	p-value
TSR						
Low	Reference		Reference		Reference	
High	2.12 (1.06–4.23)	0.033	2.19 (1.09–4.42)	0.028	2.20 (1.10–4.44)	0.027
BRAF						
Wildtype (wt)	Reference		Reference			
Mutant (mt)	2.70 (1.30–5.61)	0.008	2.11 (0.97–4.59)	0.061		NI
KRAS						
Wildtype (wt)	Reference					
Mutant (mt)	1.00 (0.49–2.04)	0.996		NI		NI
Microsatellite						
Stable (MSS)	Reference				Reference	
Instable (MSI)	2.53 (1.24–5.17)	0.011		NI	1.79 (0.81–3.99)	0.152
Age	1.01 (0.98–1.05)	0.433		NI		NI
Gender						
Male	Reference					
Female	1.65 (0.80–3.41)	0.174		NI		NI
Comorbidities						
0	Reference					
1	2.34 (0.99–5.53)	0.052				
≥ 2	2.48 (0.79–7.82)	0.121		NI		NI
Surgery						
Elective	Reference					
Acute	1.63 (0.48–5.47)	0.431		NI		NI
Tumour localisation						
Right colon	Reference					
Left colon	0.92 (0.46–1.81)	0.800		NI		NI
TNM stage						
I	Reference					
II	0.71 (0.29–1.72)	0.445		NI		NI
Differentiation						
Well/Moderate	Reference		Reference		Reference	
Poor/Undifferentiated	3.28 (1.56–6.90)	0.002	2.59 (1.16–5.76)	0.020	2.53 (1.10–5.82)	0.029
Lymph-angio invasion						
No	Reference					
Yes	1.11 (0.46–2.69)	0.814		NI		NI

Combination of the biomarkers TSR and BRAF resulted in 118 TSR-low/BRAF wildtype (wt) (58.7%), 25 TSR-low/BRAF mutant (mt) (12.4%), 49 TSR-high/BRAF wt (24.4%), 9 TSR-high/BRAF mt (4.5%). In comparison to patients with a TSR-low/BRAF wt tumour, a BRAF mutation resulted in worse DFS with a HR of 3.66, as did high TSR with a HR of 2.82 (Table 3). In patients with both prognostic factors (BRAF mt and high TSR), the risk of poor DFS increased to a HR of 4.39 (Table 3). Due to the small number of patients, potential other confounders could not be included.

**Table 3:** Association between tumour-stroma ratio combined with BRAF and KRAS mutation status and microsatellite (in)stability, and disease free survival in TNM-stage I–II colon tumours

TSR and BRAF		HR (95% CI)	p-value				
Univariable							
TSR-Low/BRAF wt	(n = 118, 58.7%)	1 (reference)					
TSR-Low/BRAF mt	(n = 25, 12.4%)	3.66 (1.47–9.09)	0.005				
TSR-High/BRAF wt	(n = 49, 24.4%)	2.82 (1.22–6.50)	0.015				
TSR-High/BRAF mt	(n = 9, 4.5%)	4.39 (1.23–15.75)	0.023				

TSR and KRAS		HR (95% CI)	p-value	TSR and KRAS		HR (95% CI)	p-value
Univariable				Multivariable*			
TSR-Low/KRAS wt	(n = 88, 43.8%)	1 (reference)		TSR-Low/KRAS wt	(n = 88, 43.8%)	1 (reference)	
TSR-Low/KRAS mt	(n = 55, 27.4%)	0.89 (0.36–2.24)	0.809	TSR-Low/KRAS mt	(n = 55, 27.4%)	0.86 (0.34–2.16)	0.748
TSR-High/KRAS wt	(n = 40, 19.9%)	2.05 (0.90–4.67)	0.088	TSR-High/KRAS wt	(n = 40, 19.9%)	1.98 (0.87–4.51)	0.106
TSR-High/KRAS mt	(n = 17, 8.5%)	2.24 (0.80–6.30)	0.125	TSR-High/KRAS mt	(n = 17, 8.5%)	2.15 (0.77–6.04)	0.146

TSR and MSI		HR (95% CI)	p-value	TSR and MSI		HR (95% CI)	p-value
Univariable				Multivariable*			
TSR-Low/MSS	(n = 115, 57.2%)	1 (reference)		TSR-Low/MSS	(n = 115, 57.2%)	1 (reference)	
TSR-Low/MSI	(n = 28, 13.9%)	3.16 (1.27–7.86)	0.013	TSR-Low/MSI	(n = 28, 13.9%)	2.12 (0.77–5.86)	0.149
TSR-High/MSS	(n = 45, 22.4%)	2.65 (1.13–6.25)	0.026	TSR-High/MSS	(n = 45, 22.4%)	2.46 (1.04–5.83)	0.041
TSR-High/MSI	(n = 13, 6.5%)	4.29 (1.36–13.47)	0.013	TSR-High/MSI	(n = 13, 6.5%)	3.31 (1.01–10.85)	0.048

*Adjusted for tumour differentiation grade

HR: hazard ratio; CI: confidence interval; wt: wild type, mt: mutant

Combining TSR with KRAS resulted in 88 TSR-low/KRAS wt (43.8%), 55 TSR-low/KRAS mt (27.4%), 40 TSR-high/KRAS wt (19.9%) and 17 TSR-high KRAS mt (8.5%). DFS was worse in patients with high TSR and KRAS wt tumours, compared to TSR-low/KRAS wt (Table 3). However, after correction for differentiation grade this association did not remain significant (Table 3).

Combination of the biomarkers TSR and MSI resulted in 115 TSR-low/MSS (57.2%), 28 TSR-low/MSI (13.9%), 45 TSR-high/MSS (22.4%) and 13 TSR-high/MSI (6.5%). In comparison to patients with a TSR-low/MSS tumour, high TSR resulted in worse DFS with a HR of 2.46, independent of the tumour differentiation grade (Table 3). TSR-low/MSI tumours did not show worse DFS compared to TSR-low/MSS tumours after correction for differentiation grade. In patients with both prognostic factors (MSI and high TSR), the risk of poor DFS increased to a HR of 3.31.

**Fig. 2:**
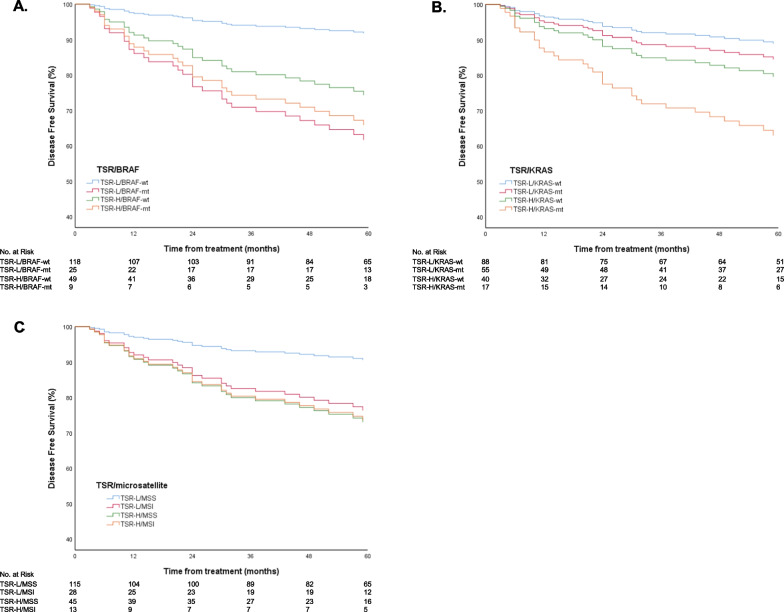
Kaplan–Meier curves of 5 year disease free survival according to groups. **A** TSR combined with BRAF-mutation state. **B** TSR combined with KRAS-mutation state. **C** TSR combined with microsatellite (in)stability

DFS-curves for these combinations are presented in Fig. 2.

## Discussion

In this study we aimed to assess whether, and to what extent, known molecular markers add prognostic value to the value of TSR alone in the identification of patients with node negative CC at high-risk of cancer recurrence. This study shows that TSR-high tumours are associated with worse DFS, independent of BRAF mutation status and microsatellite (in)stability. Judging by the higher HR for the combination of the prognostic factors TSR and BRAF compared to the HRs of these individual prognostic factors separately, prediction of a better or poorer DFS may be improved by determining both TSR and BRAF instead of BRAF alone as in current daily practice. In this study MSI also was of additional prognostic value to TSR for predicting DFS. Therefore, the adoption of TSR, BRAF and MSI as prognostic variables into current daily diagnostics might improve the risk evaluation for cancer recurrence or metastasis. Results of this study should be interpreted with caution due to the small number of events. With this retrospective study we also assessed the potential correlation between the recently found but not yet implemented marker TSR and molecular tumour markers BRAF, KRAS and MSI. Although these mutations have been shown to affect the tumour microenvironment in vivo (Janda et al. 2002; Maffeis et al. 2019; Lemieux et al. 2009; Oh et al. 2016), we did not find any relation between a tumour’s mutational status and the intratumoural stroma derived marker TSR in this study population. It should be noted that the number of mutations in this population is limited. The prognostic value of TSR in colon cancer has been demonstrated in multiple studies (Huijbers et al. 2013; Mesker et al. 2007; Park et al. 2014). For the study population in this study, the prognostic value of TSR was already determined in previous research (Strous et al. 2022).

Colorectal malignancies are heterogeneous, which can already be observed in premalignant stages. Precursor lesions can develop into carcinomas by different pathways, the suppressor pathway or the sessile serrated pathway (Pelt et al. 2018; Linden et al. 2020; Vogelaar et al. 2016; Nakanishi et al. 2019). In the sessile serrated pathway BRAF-mutations are common, sometimes in combination with MSI, while this mutation is almost never present in conventional adenomas. Mutations in the RAS-RAF-MEK-cascade, among which BRAF-mutations, induce EMT by allowing downstream effectors to promote the expression of transcription factor regulating EMT, leading to a mesenchymal phenotype of the tumour (Fridman et al. 2020; Janda et al. 2002). EMT is also promoted by intratumoural stroma, which inhibits myofibroblasts, active fibroblasts that activate the Wnt pathway and promote EMT. During this process more fibroblasts are activated, resulting in more myofibroblasts, which in turn stimulate EMT further (Conti and Thomas 2011). The mesenchymal phenotype, which is acquired by the EMT-process, is associated with poor prognosis. Both intratumoural stroma and BRAF mutations are believed to promote EMT, suggesting that a tumour yielding one, or both, of these features exhibits not only epithelial features but also mesenchymal features. This might explain the association between TSR-high tumours and tumours with a BRAF mutation, and poor prognosis. While BRAF and KRAS mutations both lead to activation of the RAS-RAF-MEK-cascade, KRAS mutations occur during tumour progression in the classical pathway or suppressor pathway while BRAF mutations are initiating mutations in the sessile serrated pathway (Nakanishi et al. 2019). Tumours with BRAF-mutations might therefore direct early to a mesenchymal phenotype while tumours with KRAS-mutations might develop without EMT (Nakanishi et al. 2019). In theory this difference might explain why BRAF-mutations are associated with poor prognosis but not KRAS-mutations. However, this result might also be due to the small size of our study cohort.

The relation between different premalignant precursor lesions of CC and microsatellite (in)stability is less straightforward. Conventional adenomas lead to microsatellite stable carcinomas via the suppressor pathway. However, the serrated pathway gives rise to both MSI and MSS carcinomas (Pelt et al. 2018). Several previous studies suggest a better prognosis for MSI tumours, even independent of presence of a BRAF-mutation (Oh et al. 2016; Farina-Sarasqueta et al. 2010; Pelt et al. 2018). But this association has been inconsistent between different studies. Morphologically, MSI tumours are more heterogeneous compared to MSS tumours. MSI tumours are associated with poorly differentiated histology, which is also seen in this study, and can present with glandular, solid and mucinous growth patterns, even combined within one tumour (Nakanishi et al. 2019; Xiao et al. 2013). Our results suggest that MSI is associated with poor prognosis, which might be related to the type of precursor lesion involved. In TSR-high tumours, a tumour expected to exhibit mesenchymal features, prognosis is poor, irrespective of MSS or MSI. However, in TSR-low tumours, a tumour in which EMT might be less present or absent, MSI is associated with worse prognosis compared to MSS. As MSI tumours are known to originate from the serrated pathway, a BRAF-mutation might be confounding in the association between MSI and poor prognosis, as these are common in the sessile serrated pathway and are associated with poor prognosis. Within this study population, MSI and BRAF-mutations were often present in the same tumour. Unfortunately, the number of inclusions in this study did not enable us to stratify MSI tumours according to BRAF-mutation.

This study was subject to a number of limitations. Because of its retrospective character, confounding could not be ruled out. Due to a relatively small number of patients, we were not able to perform adequate multivariate analyses correcting for potential confounders, as for example previously identified high-risk features, within the different combinations of TSR and mutations. Due to the small number of patients and events confidence intervals were large and HRs were less reliable. For MSI determination only one marker, mononucleotide repeat BAT 26, was used. It discriminates 99% of MSI in the Caucasian population, but makes it impossible to sub-stratify MSI tumours into MSI-low and MSI-high, which is has been shown to be discriminative in a previous study (Pawlik et al. 2004).

This study also has some important strengths. With this study we were able to analyse a combination of biomarkers composed of epithelial and micro-environmental features in a well-defined group of node negative CC patients who were not treated with adjuvant therapy. Although biomarker discovery is thriving, incorporation of biomarkers in clinical practice lags behind. The subgroup of node negative CC patients is a very interesting group for which clinicians are still not able to adequately identify patients at high-risk of recurrence. This group is expected to further increase in the near future because of screening programs. Therefore, identification and incorporation of (compound) prognostic biomarkers which help us predict the risk of recurrence in this subgroup is of paramount value, even though the benefit of chemotherapy in this subgroup of patients is still uncertain (Dotan and Cohen 2011; Eheman et al. 2016; Quasar Collaborative et al. 2007). In previous research limited benefit from chemotherapy was shown in MSI-tumours (Dotan and Cohen 2011), and TSR has been associated with chemo resistance (Strous et al. 2022). These markers can improve risk stratification of patients with node negative colon cancer, but do not seem to select patients who will benefit from current adjuvant therapies.

## Conclusion

The results of this study show that prognosis of patients with node negative CC is worse in case of a TSR-high tumour, independent of some clinical, histological and molecular markers. Combining TSR with BRAF or MSI, markers which have previously been introduced to daily diagnostics but are not yet fully implemented, seems to improve the risk evaluation for cancer recurrence or metastasis. Therefore, adopting TSR into daily diagnostics will probably be of additional value next to currently used molecular markers in risk stratification of patients with node negative colon cancer. In order to be able to present these patients a more tailor-made risk of cancer recurrence in the process of shared decision-making in the treatment of colon cancer, results of this study should be confirmed in larger future studies in which important confounders can be included.

## Data Availability

The datasets generated and/or analysed during the current study are not publicly available due to ethical concerns. Patients were included on a no objection base to conduct retrospective data studies and publish findings, but were not asked for permission to publish full encrypted data. Data are available from the VieCuri Institutional Data Access (contact via wetenschapsbureau@viecuri.nl) on reasonable request.

## Abbreviations

ASCO: American Society of Clinical Oncology
CC: Colon cancer
DFS: Disease free survival
EMT: Epithelial-to-mesenchymal transition
H&E: Haematoxylin and eosin
IQR: Interquartile range
MSI: Microsatellite instability
MSS: Microsatellite stability
mt: Mutant
NCR: Netherlands Cancer Registry
TNM: Tumour-node-metastasis
TSR: Tumour-stroma ratio
wt: Wild type
